# The protective effects of cabozantinib against high glucose-induced damages in *in vitro* renal glomerular endothelial cells model via inhibition of early growth response-1 (Egr-1)

**DOI:** 10.1080/21655979.2022.2063667

**Published:** 2022-04-20

**Authors:** Hanlu Ye, Jingjing Yan, Qiong Wang, Hui Tian, Lei Zhou

**Affiliations:** a Department of Endocrine and Metabolic Diseases, Wuhan Hospital of Traditional Chinese Medicine, Wuhan City, Hubei Province, China; b Respiratory Department Attending Surgeon, Wuhan Hospital of Traditional Chinese Medicine, Wuhan City, Hubei Province, China; c Nephrology Department Attending Surgeon, Wuhan Hospital of Traditional Chinese Medicine, Wuhan City, Hubei Province, China

**Keywords:** Cabozantinib, diabetic nephropathy (DN), human renal glomerular endothelial cells (hGECs), high glucose, oxidative stress, Egr-1

## Abstract

Cabozantinib is a tyrosine kinase inhibitor with anti-tumor activity in kidney cancer. However, the efficacy of cabozantinib in other renal diseases has never been reported. Here, we focused on exploring the effect of cabozantinib on diabetic nephropathy (DN). The biofunctions of cabozantinib in human renal glomerular endothelial cells (hGECs) under high glucose conditions have been investigated. We found that cabozantinib ameliorated high glucose-induced oxidative stress in hGECs with decreased production of mitochondrial reactive oxygen species (ROS) and increased glutathione peroxidase (GSH-PX) activity. Cabozantinib ameliorated high glucose-induced reduction in the expression of endothelial nitric oxide synthase (eNOS) and the production of nitric oxide (NO) in hGECs. It also suppressed the expression of pro-inflammatory mediators, interleukin-6 (IL-6) and monocyte chemokine protein 1 (MCP-1), against high glucose exposure in hGECs. Cabozantinib reduced the expression of early growth response-1 (Egr-1) in high glucose-treated hGECs, while Egr-1 overexpression abolished the protective effects of cabozantinib against high glucose in hGECs. In conclusion, cabozantinib protected hGECs from high glucose-induced oxidative stress, NO deficiency, and inflammation via regulating Egr-1. These findings suggest that cabozantinib might be used as an adjuvant to control DN.

## Graphical abstract

**Figure:**
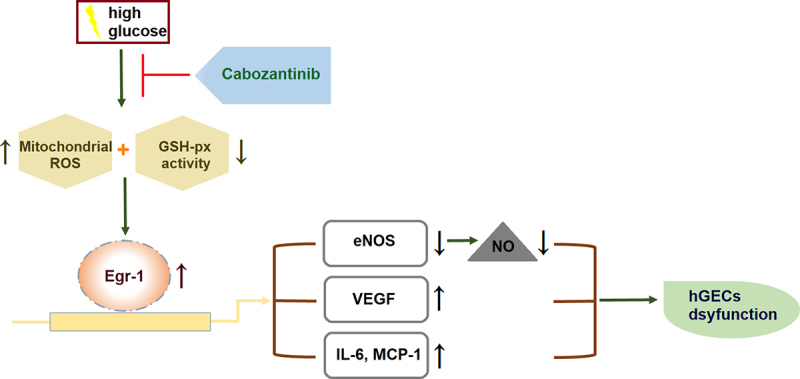

## 1. Introduction

Diabetic nephropathy (DN) is a diabetic complication causing injury to the kidney microcirculation [1]. Underlying pathogenic mechanisms have shown that hyperglycemia-caused metabolic consequences, such as accumulated Advanced Glycation End-products (AGEs) and reactive oxygen species (ROS), contribute to the pathological changes in various cell types including vascular endothelial cells (VECs), tubular epithelial cells, mesangial cells, glomerular endothelial cells (GECs), glomerular podocytes, as well as interstitial fibroblasts [2–4]. Due to the structure and function of the kidney, endothelial cells (ECs) widely exist in the kidney and its associated structures, including renal veins, venules, arteries, arterioles, and glomerular capillaries with distinctive phenotypic features [5]. The ECs have been found to play critical roles in abundant physiological functions, thus their dysfunction is implicated in the development of DN [6].

GECs covering the luminal surface of glomerular capillaries serve as a glomerular filtration barrier that is responsible for efficient filtering, secretion, and absorption [7]. GECs are particularly vulnerable to hyperglycemia-mediated injury since they are chronically exposed to high blood glucose levels [8]. GECs dysfunction is characterized by the following features: excessive generation of ROS and inflammatory mediators, metabolic changes, activation of deleterious pathways, and enhanced permeability [9]. Therefore, it is not surprising that GECs dysfunction remains a major mechanism in the pathogenesis of DN.

**Figure 1.**
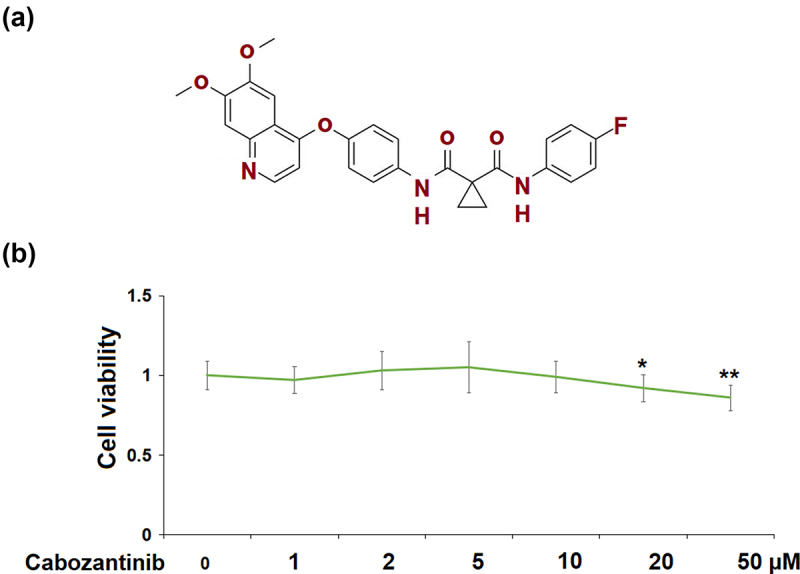
Cytotoxicity of Cabozantinib in human renal glomerular endothelial cells (hGECs). Cells were treated with Cabozantinib at various concentrations for 24 hours. (a) Molecular structure of Cabozantinib; (b) The Cell viability of Cabozantinib was determined by MTT assay, the OD value was analyzed (*, **, P < 0.05, 0.01 vs. Vehicle group).

Cabozantinib (Molecular structure is listed in Figure 1(a)) is a tyrosine kinase inhibitor that is currently approved for the treatment of various cancers, such as medullary thyroid carcinoma, and hepatocellular carcinoma [10]. It has recently been assessed for the treatment of advanced renal cell carcinoma (RCC) [11]. It improves survival outcomes in RCC patients after vascular endothelial growth factor (VEGF)‐targeted therapy [12]. However, the efficacy of cabozantinib in other renal diseases has never been reported. Here, we focused on exploring its beneficial effects on GECs dysfunction under high glucose conditions.

## 2. Materials and methods

### 2.1 Cell culture, treatment, and transduction

The protocol of this study was approved by the independent Ethical Committee of Wuhan Hospital of Traditional Chinese Medicine. Human renal glomerular endothelial cells (hGECs) (ScienCell Research Laboratories, USA) were maintained in DMEM/F12 (Gibco, USA) containing 10% fetal bovine serum (FBS) (Gibco). The hGECs were grown in humidified air with 5% CO_2_ at 37°C. Cells were exposed to high glucose (30 mM) condition, treated with or without additional cabozantinib (5 or 10 μM) for 24 h. The hGECs were transduced with Ad-viral Egr-1, followed by stimulation with high glucose (30 mM) and cabozantinib (10 μM) for 24 h.

### 2.2 3-(4,5)-dimethylthiahiazo (-z-y1)-3,5-di-phenytetrazoliumromide (MTT) assay

An MTT assay kit (Beyotime) was used to assess hGECs viability. Briefly, the hGECs (5 × 10^3^ cells/well) were seeded in a 96-well plate and incubated with MTT (5 mg/ml) for 4 h. Then 150 μl dimethyl sulfoxide (DMSO) was used to solubilize the crystals. Optical density (OD) at 490 nm was examined to calculate cell viability.

### 2.3 MitoSOX red staining

Changes in mitochondrial ROS level were determined using MitoSOX red staining reagent (MolecularProbes, USA). The hGECs were loaded with MitoSOX Red in the dark for 10 min and washed twice with PBS. Mitochondrial ROS were then assessed and captured using a fluorescence microscope, followed by the determination of fluorescence intensity using Image J software [13].

### 2.4 Glutathione Peroxidase (GSH-PX) activity detection

GSH-PX activity in hGECs was assessed by colorimetric method using a GSH-PX assay kit (Jiancheng Bioengineering Institute, Nanjing, China). Optical density (OD) at 412 nm was recorded using a SpectraMax microplate spectrophotometer (Molecular Devices, Sunnyvale, CA, USA) to assess GSH content.

### 2.5 RT-qPCR

Total RNAs obtained from hGECs using TRIzol reagent (Invitrogen) were subsequently reversely transcribed into cDNAs using a cDNA synthesis kit (Bio-Rad, USA). Afterward, the cDNAs were amplified using PCR with SYBR Green Real-time PCR Master Mix (TOYOBO, Japan). The relative expressions of Egr-1, VEGF, IL-6, MCP-1, eNOS were calculated with the 2^−ΔΔCt^ method relative to the internal control GAPDH [14]. The following primers were used: IL-6: 5’-ATGAACTCCTTCTCCACAAGCGC-3’ (Forward) and 5’-GAAGAGCCCTCAGGCTGGACTG-3’ (Reverse); VEGF: 5’-GAGGAGCAGTTACGGTCTGTG-3’ (Forward) and 5’-TCCTTTCCTTAGCTGACACTTGT-3’ (Reverse); MCP-1: 5’-CTCATAGCAGCC CCTTATTCC-3’ (Forward) and 5’-GATCACAGCTTCTTTGGGACACT-3’ (Reverse); Erg-1: 5’-GGTCAGTGCCTAGTGAGC-3’ (Forward) and 5’-GTGCCGCTGAGTAAATGGGA-3’ (Reverse); eNOS: 5’-TGATGG GAAGCGAGTGAAG-3’ (Forward) and 5’-ACTCATCCATAACAGACCC-3’ (Reverse); GAPDH: 5’-CATCATCCCTGCCTCTACTGG-3’ (Forward) and 5’-GTGG TTCGCTGTTGAAGTC-3’ (Reverse).

### 2.6 Western blot

Western blot was performed to assess the expression levels of eNOS, Egr-1, and VEGF in hGECs [15]. Briefly, cellular lysates extracted from hGECs was separated by 10% sodium dodecyl sulfate-polyacrylamide gel electrophoresis (SDS-PAGE) gels, transferred onto polyvinylidene fluoride (PVDF) membranes, and incubated with primary antibodies and HRP-labeled secondary antibodies (eNOS: #32,027; Egr-1: #4154; GAPDH: # 5174; secondary antibodies #7074 and #7076, Cell Signaling Technology, USA). The protein bands were visualized by Quantity One software (Bio-Rad, USA).

### 2.7 Diaminofluorescein-2 diacetate (DAF-2 DA) staining

The NO level in hGECs was assessed using DAF-2 DA (Sigma-Aldrich, USA) staining for 45 min at 37°C. The fluorescence of product triazolofluorescein (DAF-2 T) was detected and the fluorescence intensity was analyzed using ImageJ software.

### 2.8 Enzyme-linked immunosorbent assay (ELISA)

The levels of IL-6, MCP-1, and VEGF in the cell culture fluid of hGECs were measured using the commercial ELISA kits (R&D Systems) according to the manufacturer’s instructions.

### 2.9 Statistical analysis

The SPSS 19.0 statistical software (SPSS Inc., USA) was used for data analysis. All the results were expressed as means ± SD. One-way ANOVA was used for comparisons among multi-group data, followed by the Tukey’s post-hoc test. A *p*-value < 0.05 was considered significant.

## 3. Results

In this study, we established an *in vitro* DN model using HG- stimulated hGECs to explore the potential benefits of cabozantinib on DN. Our results reveal that cabozantinib treatment ameliorated HG-induced oxidative stress and expression of VEGF and pro-inflammatory mediators. Furthermore, we found that cabozantinib rescued the HG-induced reduction in the expression of eNOS and production of NO. Importantly, we demonstrate that the protective effects of cabozantinib on HG- stimulated hGECs were mediated by Erg-1.

### 3.1 Cytotoxicity of cabozantinib in hGECs

In Figure 1(b), cell viability of hGECs did not differ between the control group and cabozantinib group at various concentrations (1, 2, 5, 10 μM), except for the 20 and 50 μM cabozantinib groups. In the following studies, 5 and 10 μM were used.

### 3.2 Cabozantinib ameliorated high glucose-induced oxidative stress in hGECs

**Figure 2.**
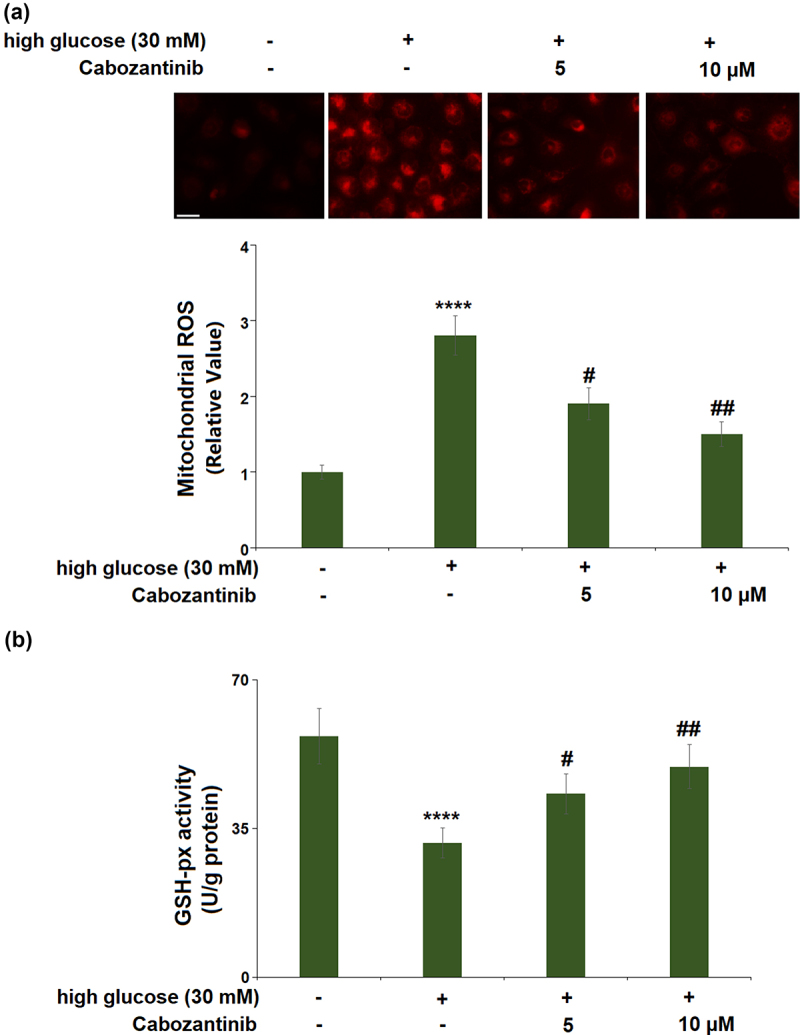
Cabozantinib ameliorated high glucose-induced oxidative stress in hGECs.

For mitochondrial ROS production (Figure 2(a)), high glucose induced a 2.8-fold increase, whereas 5 or 10 μM cabozantinib showed significant reductions (0.68-fold and 0.54-fold). For the GSH-px activity (Figure 2(b)), a 0.56-fold reduction was observed in the high glucose group, whilst cabozantinib (5 or 10 μM) caused 1.37- and 1.57-fold increases as compared to the high glucose group.

### 3.3 Cabozantinib ameliorated high glucose-induced reduction in the expression of eNOS and production of NO in hGECs

**Figure 3.**
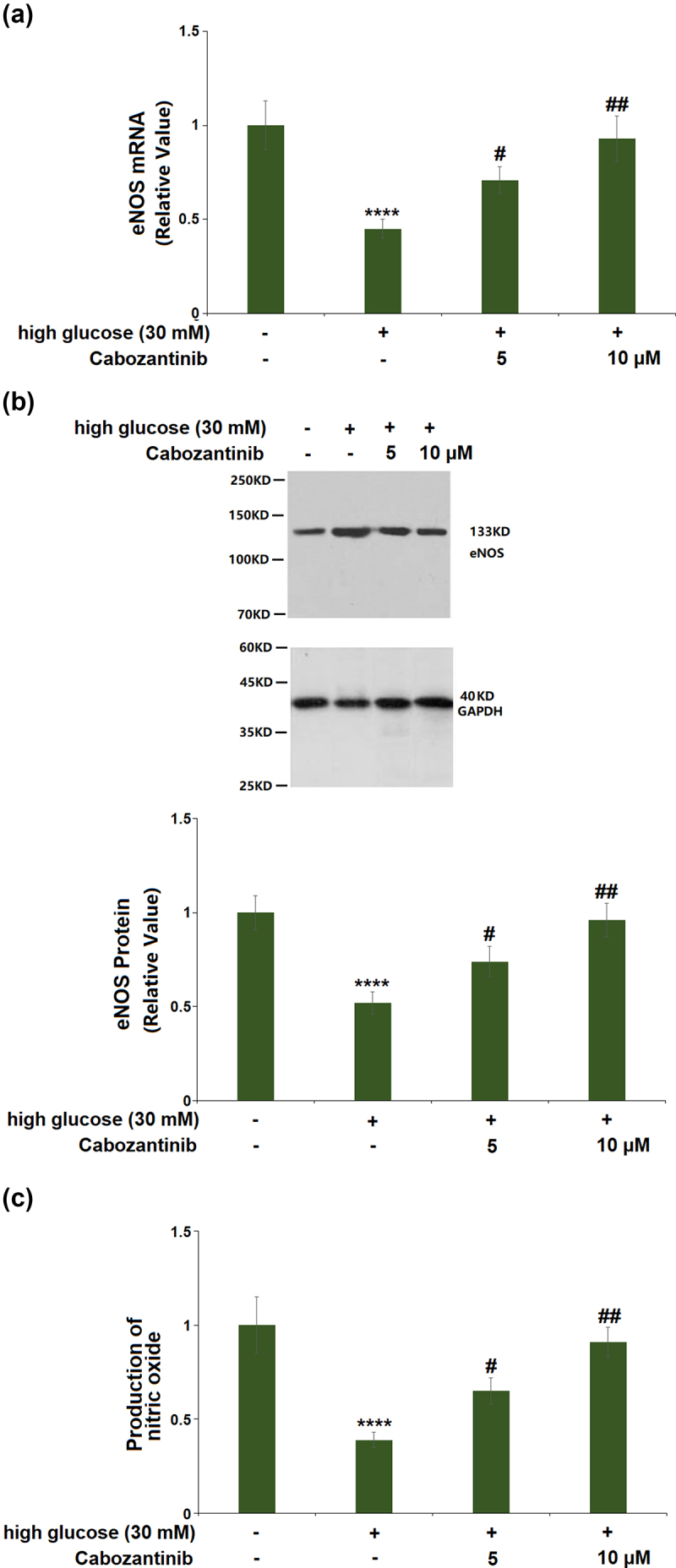
Cabozantinib ameliorated high glucose-induced reduction in the expression of eNOS and the production of NO in hGECs. (a). mRNA of eNOS; (b). Protein levels of eNOS; (c). Production of nitric oxide (****, P < 0.0001 vs. Vehicle group; #, ##, P < 0.05, 0.01 vs. high glucose group).

As shown in Figure 3(a), the mRNA of eNOS was decreased by 0.45-fold by treatment with high glucose medium, and the decrease was elevated by cabozantinib (5 or 10 μM) with a 1.58- or 2.07-fold change. Consistent with the PCR results, western blot showed that the decreased protein level of eNOS (0.52-fold) in the high glucose group was increased by 1.42- or 1.85-fold after treatment with 5 or 10 μM cabozantinib (Figure 3(b)). Also, DAF-2 DA staining showed that the production of NO had a 0.39-fold reduction, which could be attenuated by 5 or 10 μM cabozantinib with a 1.67- or 2.33-fold change (Figure 3(c)).

### 3.4 Cabozantinib inhibited the expression of VEGF against high glucose in hGECs

**Figure 4.**
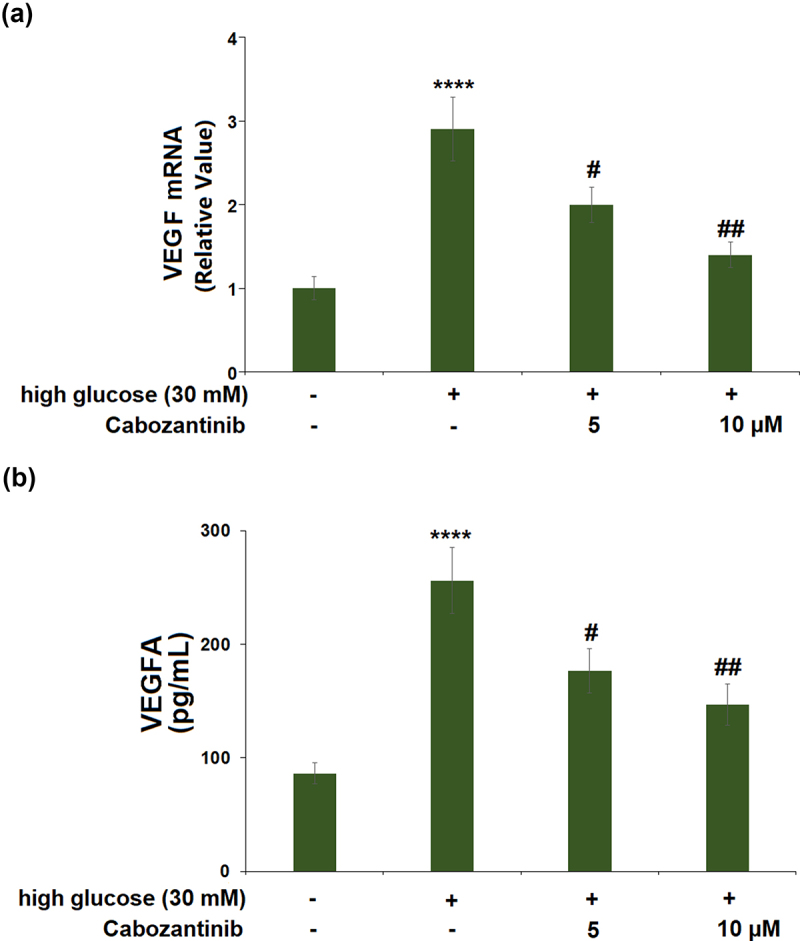
Cabozantinib inhibited the expression of VEGF against high glucose in hGECs. (a) mRNA level of VEGF; (b) protein level of VEGF measured by ELISA (****, P < 0.0001 vs. Vehicle group; #, ##, P < 0.05, 0.01 vs. high glucose group).

High glucose stimulation caused a significant 2.9-fold increase in the mRNA level of VEGF. Treatment with 5 or 10 μM cabozantinib caused 0.69-fold and 0.48-fold decreases in the VEGF mRNA level, compared with cells exposed to high glucose alone (Figure 4(a)). Meanwhile, ELISA results demonstrate that exposure to high glucose led to the protein levels of VEGF being increased from 86.3 to 256.1 pg/mL, which was then reduced to 176.7 and 146.5 pg/mL by 5 or 10 μM cabozantinib with a 0.73- or 0.54-fold change (Figure 4(b)).

### 3.5 Cabozantinib suppressed the expression of pro-inflammatory mediators against high glucose in hGECs

**Figure 5.**
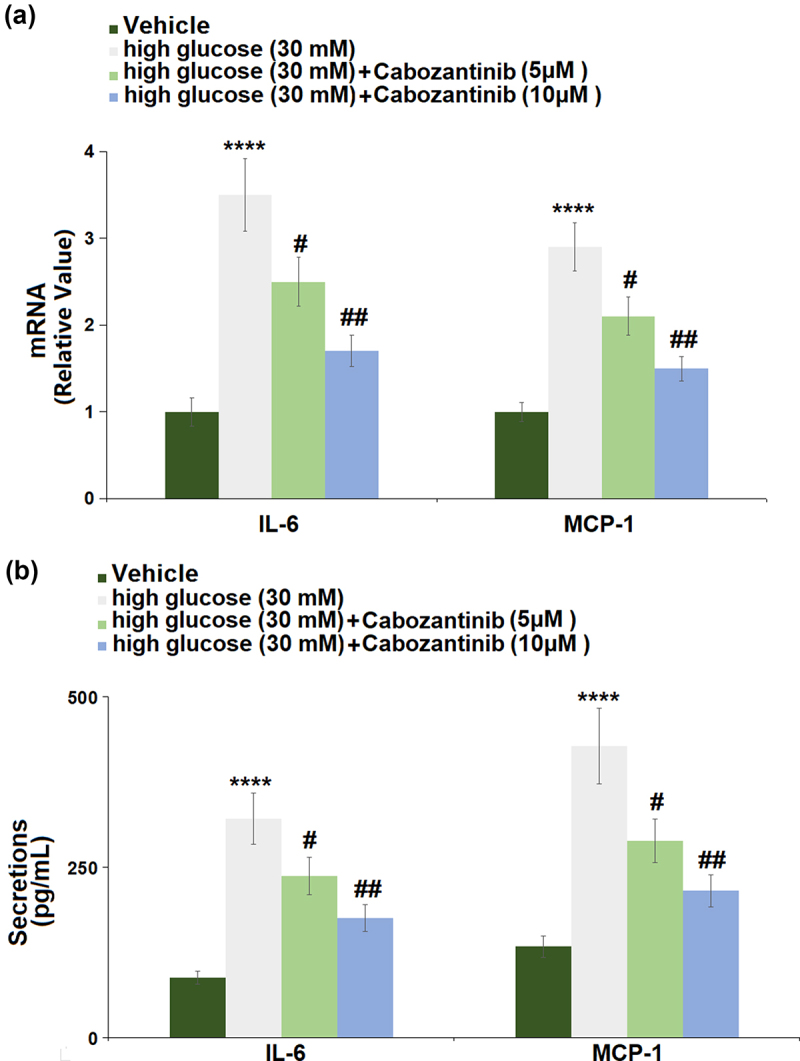
Cabozantinib suppressed the expression of pro-inflammatory mediators against high glucose in hGECs. (a). mRNA of IL-6 and MCP-1; (b). Secretions of IL-6 and MCP-1 (****, P < 0.0001 vs. Vehicle group; #, ##, P < 0.05, 0.01 vs. high glucose group).

Figure 5(a) shows the changes in mRNA levels of IL-6 and MCP-1 in hGECs. The hGECs grown in high glucose medium showed a significant increase in the mRNA levels of IL-6 (3.5-fold) and MCP-1 (2.9-fold), which could be remarkably abrogated by 5 or 10 μM cabozantinib. Figure 5(b) shows the changes in secretion levels of IL-6 and MCP-1, suggesting that high glucose-induced increases in the secretion levels of IL-6 (3.65-fold) and MCP-1 (3.20-fold) were attenuated by 5 or 10 μM cabozantinib.

### 3.6 Cabozantinib reduced the expression of Egr-1 against high glucose in hGECs

**Figure 6.**
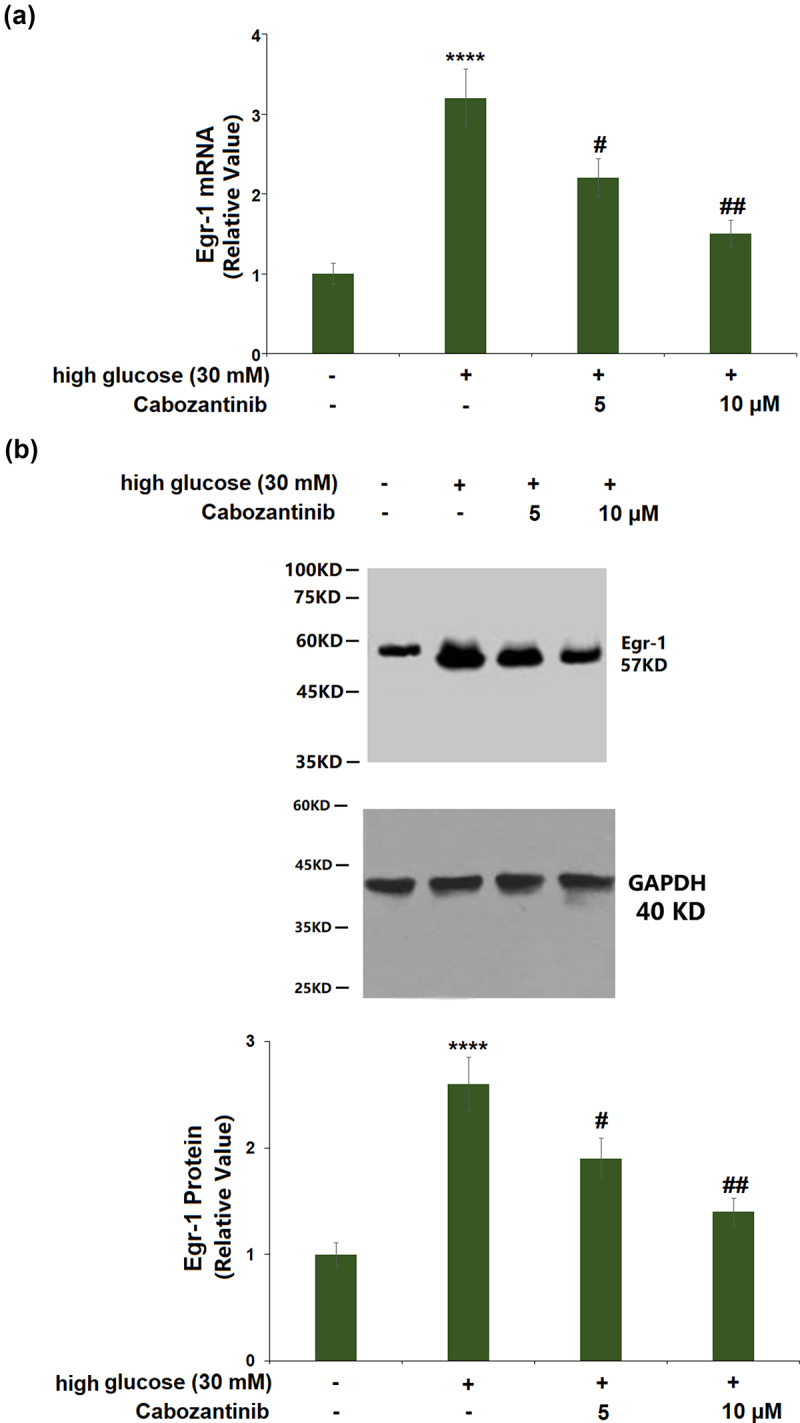
Cabozantinib reduced the expression of Egr-1 against high glucose in hGECs. (a). mRNA of Egr-1; (b). Protein of Egr-1 (****, P < 0.0001 vs. Vehicle group; #, ##, P < 0.05, 0.01 vs. high glucose group).

Figure 6(a) shows a 3.2-fold increase in the mRNA of Egr-1 in high glucose-induced hGECs. Cabozantinib (5 or 10 μM)-treated hGECs showed a 0.69- or 0.47-fold reduction in the mRNA of Egr-1 when compared to hGECs exposed to high glucose alone. Similarly, the significantly increased protein level of Egr-1 (2.6-fold) in high glucose-induced hGECs was reduced by cabozantinib (5 or 10 μM) with a 0.73- or 0.54-fold change (Figure 6(b)).

### 3.7 Overexpression of Egr-1 abolished the protective effects of cabozantinib against high glucose in hGECs

**Figure 7.**
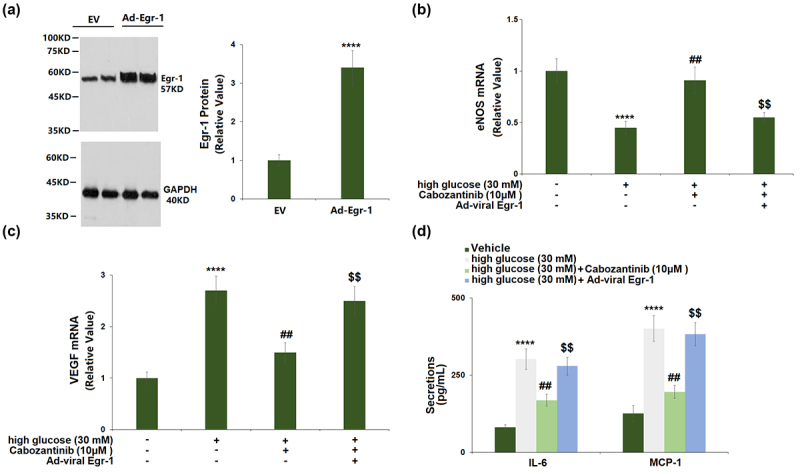
Overexpression of Egr-1 abolished the protective effects of Cabozantinib against high glucose in hGECs. Cells were transduced with Ad-viral Egr-1, followed by stimulation with high glucose (30 mM) with Cabozantinib (10 μM) for 24 hours. (a). Western blot analysis revealed successful overexpression of Egr-1; (b). mRNA of eNOS; (c). mRNA level of VEGF; (d). Secretions of IL-6 and MCP-1 (****, P < 0.0001 vs. Vehicle group; ##, P < 0.01 vs. high glucose group; $$, P < 0.01 vs. High glucose+Ad-Egr-1 group).

The transduction efficiency assay showed that Egr-1 expression was markedly increased by 3.4-fold in cells transduced with Ad-viral Egr-1, confirmed by Western blot (Figure 7(a)). Transduction with Ad-viral Egr-1 resulted in a significant decrease in eNOS mRNA (0.60-fold) and a significant increase in VEGF mRNA (1.67-fold) when compared to the cells transduced with control Ad-viral in the presence of 10 μM cabozantinib (Figures 7(b-c)). In addition, the cabozantinib-caused reduction in the secretion of IL-6 and MCP-1 was attenuated by Ad-viral Egr-1 transduction (Figure 7(d)).

## 4. Discussion

ECs dysfunction is characterized by several metabolic abnormalities: increased mitochondrial ROS overproduction, decreased NO bioavailability, accumulation of inflammatory factors, and extracellular matrix (ECM) proteins [9]. Previous literatures have confirmed the vital role of mitochondria dysfunction in the progression of DN [16]. Amongst the changes in mitochondria, the accumulation of mitochondrial ROS is the pivotal issue during persistent hyperglycemia [17]. Overproduction of ROS in a hyperglycemic environment causes persistent oxidative damage, giving way to mitochondrial dysfunction, which further increases the production of ROS, thereby forming a vicious cycle in mitochondria [18,19]. We found that cabozantinib ameliorated high glucose-induced oxidative stress in hGECs with decreased production of mitochondrial ROS and increased GSH-PX activity.

NO, a free radical produced in almost all tissues, is a paracrine mediator exerting a variety of biological actions in ECs [20]. Particularly, NO acts as an important regulator of renal function and controls medullary blood flow, glomerular ultrafiltration coefficient, and vascular tone [21,22]. NO production is transcriptionally or post-translationally regulated by three isoforms of NOS: eNOS, inducible NOS (iNOS), and neuronal (nNOS) [23]. The eNOS gene has been considered to be involved in the progression of DN. In a diabetic state, ECs dysfunction results in reduced activation of eNOS, which in turn reduces the generation and bioavailability of NO [5]. In addition, the eNOS uncoupling in ECs dysfunction also leads to overproduction of ROS, which further causes oxidative and inflammatory damage by upregulating adhesion molecules and proinflammatory mediators [24]. Our results show that cabozantinib ameliorated high glucose-induced reduction in the expression of eNOS and the production of NO in hGECs.

ECs dysfunction also leads to an uncoupling of the VEGF-NO axis, resulting in the enhanced simulative effects of VEGF on cell proliferation and inflammatory response in ECs [25,26]. Therefore, in response to hyperglycemia, VEGF is increased in ECs and has a deleterious role in DN [27]. Here, we found that VEGF expression was induced by high glucose in hGECs, which could be reversed by cabozantinib. Cabozantinib also suppressed the expressions of pro-inflammatory mediators, IL-6 and MCP-1, against high glucose in hGECs.

Egr-1 is a member of the EGR family of transcription-regulatory factors [28]. Egr-1 is widely expressed in various renal cell types including renal ECs, tubular fibroblasts, and glomerular mesangial cells [29]. Recent studies indicate Egr-1 contributes to the development of various kidney diseases by promoting renal inflammation and fibrosis [30]. Notably, Egr-1 has been demonstrated to be involved in the progression of DN. For instance, it mediates the protective effect of the long noncoding RNA NONHSAG053901 on diabetic nephropathy through regulating renal inflammation [31]. Klotho prevents cell proliferation and suppresses the excessive extracellular matrix (ECM) production in high glucose-treated human mesangial cells, which is partially attributed to Egr-1 downregulation [32]. Lipoxins reverse the progression of diabetic kidney disease with improved collagen deposition, mesangial expansion, and albuminuria through regulating the Egr-1 network [33]. Here, we found that cabozantinib reduced the expression of Egr-1, while its overexpression abolished the protective effects of cabozantinib against high glucose in hGECs, implying that the effects of cabozantinib were mediated by Egr-1.

However, the limitation of the current study should be mentioned. We only examined the beneficial effects of cabozantinib against high glucose-induced hGECs injury in an *in vitro* hGECs cell model. It should be noted that the pathological mechanism of DN is complicated. A diversity of risk factors including genetics and aging are reportedly involved. Further *in vivo* investigations using animal models are necessary to verify the pharmacological function of cabozantinib in DN. Secondly, multiple signaling pathways have been reported to participate in the initiation and development of DN. In this study, we report that the beneficial effects of cabozantinib are mediated by Egr-1. However, it is still unknown whether the function of Egr-1 is direct or indirect. A previous study showed that Egr-1 deficiency attenuates renal inflammation in Egr1−/− mice. Egr-1 deficiency inhibited the expressions of TNF-α, MCP-1, and NLRP3 inflammasome. Furthermore, decreased NF-κB activity is also observed in Egr1−/− mice [34]. Egr-1 usually acts with the transcription factor NF-κB in synergy for the transcription of pro-inflammatory mediators [35]. Therefore, the interaction between Egr-1 and NF-κB in DN needs to be further investigated.

## Conclusion

In conclusion, cabozantinib protected hGECs against oxidative stress, NO deficiency, and inflammation in response to high glucose exposure via regulating Egr-1. These findings suggest that the administration of cabozantinib might act as adjuvant therapy to the management of DN in patients with diabetes.

## Data Availability

Data of this study are available upon reasonable request to the corresponding authors.
